# The Impact of Lifetime Alcohol and Cigarette Smoking Loads on Amyotrophic Lateral Sclerosis Progression: A Cross-Sectional Study

**DOI:** 10.3390/life11040352

**Published:** 2021-04-17

**Authors:** Aliona Cucovici, Andrea Fontana, Andrei Ivashynka, Sergio Russo, Valentina Renna, Letizia Mazzini, Ileana Gagliardi, Jessica Mandrioli, Ilaria Martinelli, Vitalie Lisnic, Dafin Fior Muresanu, Michele Zarrelli, Massimiliano Copetti, Maurizio A. Leone

**Affiliations:** 1 Department of Medical and Surgical Sciences, University of Foggia, 71122 Foggia, Italy; aliona.cucovici@unifg.it; 2 Neurology Unit, Fondazione IRCCS Casa Sollievo della Sofferenza, 71013 San Giovanni Rotondo, Italy; a.ivashynka@operapadrepio.it (A.I.); valerenna87@gmail.com (V.R.); m.zarrelli@operapadrepio.it (M.Z.); 3 Unit of Biostatistics, Fondazione IRCCS Casa Sollievo della Sofferenza, 71013 San Giovanni Rotondo, Italy; a.fontana@operapadrepio.it (A.F.); m.copetti@operapadrepio.it (M.C.); 4 Department of Health Sciences, University of Eastern Piedmont, 28100 Novara, Italy; 5 ICT Innovation & Research Unit, Fondazione IRCCS Casa Sollievo della Sofferenza, 71013 San Giovanni Rotondo, Italy; s.russo@operapadrepio.it; 6 Department of Neurology and ALS Centre, University of Piemonte Orientale, Maggiore della Carità Hospital, 28100 Novara, Italy; letizia.mazzini@uniupo.it (L.M.); ileanagagliardi91@gmail.com (I.G.); 7 Neurology Unit, Department of Neurosciences, Azienda Ospedaliero Universitaria di Modena, 41125 Modena, Italy; mandrioli.jessica@aou.mo.it (J.M.); martinelli.ilaria88@gmail.com (I.M.); 8 Department of Neurology, State University of Medicine and Pharmacy “Nicolae Testemitanu”, 2004 Chisinau, Moldova; lisnicv@yahoo.com; 9 Department of Clinical Neurosciences, University of Medicine & Pharmacy, Cluj-Napoca, Romania and “RoNeuro” Institute for Neurological Research and Diagnostic, 400000 Cluj-Napoca, Romania; office@ssnn.ro

**Keywords:** amyotrophic lateral sclerosis, smoking, alcohol drinking, disease progression rate, prognosis, questionnaire

## Abstract

Background—Amyotrophic lateral sclerosis (ALS) is a devastating and untreatable motor neuron disease; smoking and alcohol drinking may impact its progression rate. Objective—To ascertain the influence of smoking and alcohol consumption on ALS progression rates. Methods—Cross-sectional multicenter study, including 241 consecutive patients (145 males); mean age at onset was 59.9 ± 11.8 years. Cigarette smoking and alcohol consumption data were collected at recruitment through a validated questionnaire. Patients were categorized into three groups according to ΔFS (derived from the ALS Functional Rating Scale-Revised and disease duration from onset): slow (*n* = 81), intermediate (80), and fast progressors (80). Results—Current smokers accounted for 44 (18.3%) of the participants, former smokers accounted for 10 (4.1%), and non-smokers accounted for 187 (77.6%). The age of ALS onset was lower in current smokers than non-smokers, and the ΔFS was slightly, although not significantly, higher for smokers of >14 cigarettes/day. Current alcohol drinkers accounted for 147 (61.0%) of the participants, former drinkers accounted for 5 (2.1%), and non-drinkers accounted for 89 (36.9%). The log(ΔFS) was weakly correlated only with the duration of alcohol consumption (*p* = 0.028), but not with the mean number of drinks/day or the drink-years. Conclusions: This cross-sectional multicenter study suggested a possible minor role for smoking in worsening disease progression. A possible interaction with alcohol drinking was suggested.

## 1. Introduction

Amyotrophic lateral sclerosis (ALS) is an intractable neurodegenerative disease that is characterized by the progressive degeneration of motor neurons. The main clinical predictors of progression are age, site of onset, diagnostic delay, and the ALS Functional Rating Scale-Revised (ALSFRS-R) baseline score [1]. The role of some potentially modifiable lifestyle factors, such as cigarette smoking and alcohol consumption, have been studied for their possible impact on the risk of developing ALS (susceptibility) [2,3], but not as much for their possible impact on ALS progression. Cigarette smoking was found to increase the susceptibility to ALS in most studies [4,5,6,7], although some aspects are still unclear, such as the absence of a dose-dependency [8]. In contrast, the results for alcohol intake are more controversial, showing an increased [9], or a reduced risk [10], or no association [11,12]. Since the risk factors for progression may not necessarily match those for susceptibility to the disease [13], we aimed to assess a possible role of lifetime smoking and alcohol drinking on ALS progression.

## 2. Materials and Methods

The study was designed as a cross-sectional multicenter study. It was conducted in three centers in Italy (San Giovanni Rotondo, Coordinating Center, Novara, and Modena), one in the Republic of Moldova (Chisinau), and one in Romania (Cluj-Napoca). The study was approved by the Institutional Review Boards of the coordinating center (N96/CE/2016) and the other four centers. Written informed consent was obtained from all participants. The Strengthening the Reporting of Observational Studies in Epidemiology (STROBE) cross-sectional reporting guidelines [14] were used as the reference for reporting the study (Supplemental File 1).

### 2.1. Patients

Patients of both sexes were consecutively enrolled from October 2016 to January 2020, in different periods in each center. The inclusion criteria were as follows: (1) age higher than 18 years old, (2) diagnosis according to the El Escorial criteria [15], and (3) consecutive in- and out-patients with a new (incident) or already present (prevalent) clinical diagnosis of ALS. The exclusion criteria were as follows: (1) patients with a tracheostomy or receiving mechanical ventilation, (2) patients with percutaneous endoscopic gastrostomy, and (3) patients who did not sign the informed consent form.

### 2.2. Data Collection and Disease Progression Assessment

For each patient, we collected demographic (date of birth, gender, education) and clinical variables (date of onset and diagnosis, site of onset, diagnostic category according to the El Escorial criteria, BMI, forced vital capacity (FVC), therapy). Interviews were conducted during the clinical visit by interviewers that were blinded to the patients’ clinical history and neurological status. Disease severity was estimated through the ALSFRS-R, which evaluates the severity of the disease through a 12-item questionnaire [16]. The rate of disease progression (ΔFS score) at recruitment was calculated by dividing the ALSFRS-R total score by the symptom duration (months) by applying the following formula: ΔFS = 48 − (total ALSFRS-R at recruitment)/symptom duration in months [17]. The date of disease onset was determined based on subjective complaints, information confirmed by relatives, and clinical charts.

### 2.3. Exposure Assessment

Cigarette smoking and alcohol consumption data were collected at recruitment through the “Questionnaire of Lifestyle,” which is part of the European Prospective Investigation into Cancer and Nutrition project (EPIC) study [18,19]. We defined three categories of smoking status at recruitment in relation to the disease onset: non-smokers were those who had smoked <100 cigarettes up to the time of the interview [20] or stopped smoking at least six months before the disease onset; current smokers were those who had smoked ≥100 cigarettes and were still smoking at the time of the interview, or within six months of the interview; former smokers were those who had smoked ≥100 cigarettes and had stopped smoking after disease onset, but at least six months prior of recruitment.

All smokers were asked to state the age when they started and quit smoking (if appropriate), and to quantify the number of cigarettes smoked per day at the ages of 20, 30, 40, 50, 60, and ≥70 years up to the participants′ current age. For each age period, we calculated the mean number of cigarettes smoked per day based on the questionnaire information and the number of years spent smoking (i.e., smoking duration). The cigarette smoking duration (years) was calculated as the difference between the age at recruitment or smoking cessation and age when the participant started smoking. According to Peters et al. [7] we estimated the smoking intensity (cigarettes per day) as the weighted mean of the number of cigarettes smoked per day during different age periods, with weights equal to the smoking duration within each age period. Pack-years (a measure of lifetime smoking load) was calculated by dividing the smoking intensity by 20 and multiplying the result by the smoking duration (in years).

Similarly, detailed information was obtained regarding alcohol consumption during different age periods up to the participants′ current age. In relation to the disease onset, we defined non-drinkers as those who had drunk less than one standard alcoholic drink/month up to the time of the interview, or had stopped drinking at least six months before the disease onset; current drinkers were those who had drunk more than one standard alcoholic drink/month and were still drinking at the time of the interview, or within six months of the interview; former drinkers were those who had drunk more than one standard alcoholic drink/month and had stopped drinking after disease onset, but at least six months prior to recruitment. All drinkers were asked to state the age when they started and quit drinking (if appropriate), and to state the number of alcoholic drinks per day by type of beverage (wine, beer, and spirits) at the age of 20, 30, 40, 50, 60, and ≥70 years up to the participants′ current age. An Italian standard alcoholic drink (standard alcoholic unit) contains approximately 12 g of pure ethanol [21], corresponding to a small glass of wine (125 mL), a can of beer (330 mL), or a shot of spirits (40 mL). Analogous with the measures obtained for smoking, we calculated the drinking intensity (drinks/day) as the weighted mean number of standard alcoholic units per day during different age periods with weights equal to the number of years spent drinking (i.e., drinking duration) within each age period for each type of beverage, and in total (aggregating all types of drinks). Drink-years (a measure of the cumulative lifetime alcohol drinking load) was calculated by multiplying the drinking intensity by the drinking duration (in years).

### 2.4. Validation and Administration of the Questionnaire

The questionnaire was designed in Italian, then translated into Romanian by a native Romanian speaker, and backtranslated by an Italian native speaker. In two sites, two raters, previously trained in the use of the questionnaire and blinded to the patients’ clinical status, interviewed patients in a dedicated room. To evaluate the reliability of the questionnaire (inter-rater agreement), two pairs of raters interviewed healthy people or patients with neurological diseases before the study started (40 in Chisinau and 25 in San Giovanni Rotondo). The sequence of interviews was randomized and the randomization list was concealed. Each rater did the interviews on at least one day and no more than seven days apart; this was considered a sufficient time window for the subject being unable to remember his or her answers and not to change his or her consumption habits. Agreement between the two raters for consumption (yes/no) was calculated using Cohen′s kappa statistics [22] and was 0.88/1.0 for smoking/drinking in Chisinau and 0.92/0.95 in San Giovanni Rotondo. Agreement for continuous variables was determined with the intraclass correlation coefficient [23], and differed for the different variables in Chisinau (0.57/1.0) and San Giovanni Rotondo (0.63/1.0).

### 2.5. Statistical Analysis

The patients′ characteristics are reported as mean ± standard deviation, or median along with range, depending on their distribution, and with absolute and relative frequencies (percentages) for continuous and categorical variables, respectively. The normality of the continuous variables’ distributions was checked using Q-Q plots and the Shapiro–Wilk test. In the presence of right-skewed continuous variables, statistical analyses were performed on log values. Comparisons between two categorical variables were assessed using chi-square or Fisher exact tests (as appropriate), whereas comparisons between a continuous and a categorical variable were assessed using univariable and multivariable ANOVA models. Pairwise comparisons between groups of the categorical variables were performed (using the ANOVA models) and, if necessary, least-square means of the dependent variable (along with their 95% confidence interval (CI)) were estimated for each level of the categorical variable. The standardized mean difference was further reported to quantify, from a clinical perspective, the differences in investigated variables between groups and was computed as the average of all possible standardized mean differences across pairwise comparisons. The correlation between two continuous variables was assessed using Pearson’s correlation coefficient. To visually assess the relationship between the measures of the intensity (cigarettes or drinks per day), cumulative lifetime load (pack- or drink-years), and the duration of consumption as independent variables and ΔFS as the dependent variable, boxplots and scatterplots with fitted regression lines were depicted in a plot matrix. To detect all the clinical, demographical, pathological, treatment, and lifestyle variables, which were mostly associated with ΔFS, the conditional random forest (RF) algorithm [24] with 100,000 trees was used. The RF is a popular machine learning tool that assesses the relationship between a dependent variable and a set of covariates in a (nonparametric) tree-based fashion. An important feature of an RF is that it provides a rapidly computable internal measure of variable importance (VIMP) that can be used to rank variables. Moreover, the VIMP produced by a conditional RF is not affected by the correlation structure of all the included covariates. Formally, a VIMP of a specific covariate is defined as the sum of the decrease in prediction error values when a tree of the forest is split by that covariate. The more a tree relies on a variable to make predictions, the more important it is for that tree. The relative importance is the VIMP divided by the highest VIMP value. To better understand the marginal relationship between the value of each “important” variable (i.e., with VIMP > 0) and log(ΔFS), a scatterplot of the accumulated local effects [25], which were estimated from the fitted conditional RF, was provided. In addition, the joint relationship (i.e., interaction) between smoking and alcohol intensity effects on log(ΔFS) was investigated using a partial dependence plot [25].

A two-sided *p*-value < 0.05 was considered to represent statistical significance. All statistical analyses were performed using SAS Release 9.4 (SAS Institute, Cary, NC, USA). Conditional random forests and plots were performed using R Foundation for Statistical Computing (version 3.6, packages: party, GGally, iml).

## 3. Results

We recruited 241 patients, 145 men and 96 women, with a sex ratio of 1.5:1. Onset was in the spinal district in 187 patients (77.6%) and bulbar in 54 patients (22.4%). The mean age was 59.9 ± 11.8 years at onset and 62.4 ± 11.0 at recruitment. The median time that elapsed between disease onset and recruitment was 20 months (range 1.7–273). According to the El Escorial criteria, 74 (30.7%) patients were categorized as definite, 77 (32.0%) as probable, 55 (22.8%) as possible, and 35 (14.5%) as suspected in terms of their ALS diagnosis. Other demographic and clinical characteristics are shown in Table 1. The ALSFRS-R scores ranged from 10 to 48, with a mean of 34.9 ± 8.3. The ΔFS score ranged from 0 to 5.3, with a median of 0.56 (IQR: 0.25–1.05). Patients were categorized into tertiles according to the ΔFS distribution: (I) ≤0.333 (slow disease progression rate), (II) 0.334–0.875 (intermediate progression rate), and (III) >0.875 (fast progression rate).

Table 1 shows the clinical characteristics according to the ΔFS tertiles. As expected, slow progressors were younger at disease onset and recruitment, were less likely to have a bulbar onset, and had a better FVC. The El Escorial categories were associated with the progression rate; however, since some of the “suspected ALS” patients may turn out to eventually not have ALS, we made a sensitivity analysis excluding “suspected ALS.” The statistical significance did not substantially change (*p* = 0.010).

### 3.1. Smoking

**Figure 1:**
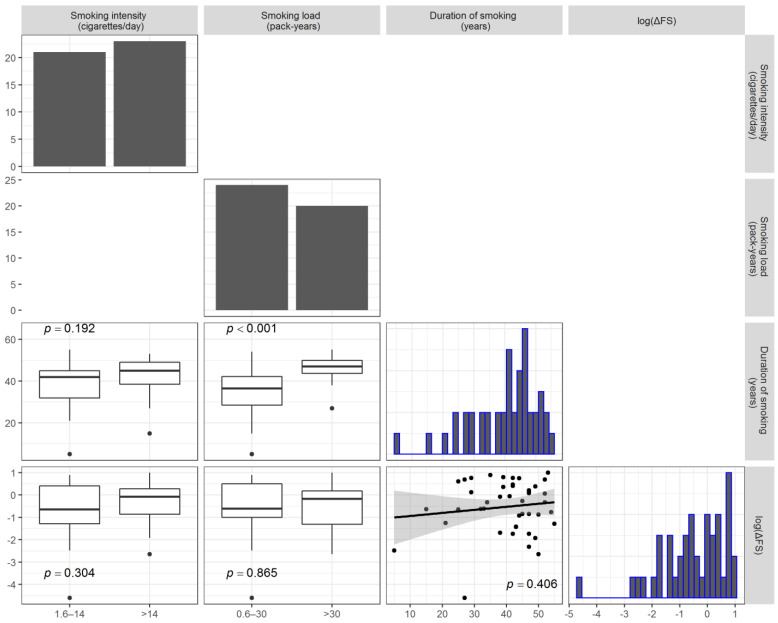
Plot matrix depicting the pairwise associations between the smoking intensity (cigarettes/day), smoking load (pack-years), duration of smoking, and log-transformed ΔFS (lower diagonal elements). Comparisons with the smoking loads are reported as boxplots, whereas the correlation between the log-transformed ΔFS and duration of smoking is reported as a scatterplot with a fitted regression line. The distribution of each variable considered is reported as a bar chart or histogram along the diagonal. Only current smokers were considered to produce the analysis results presented here.

Current smokers accounted for 44 (18.3%) of the participants, 187 (77.6%) were non-smokers, and 10 (4.1%) were former smokers. No patient started smoking after their ALS diagnosis. No difference was found for the status and modalities of smoking (Table 1). Table 2 shows the unadjusted comparisons of clinical variables according to the intensity of smoking (cigarettes/day) categories. Former smokers were excluded from the analysis. Never-smokers had a significantly higher age at ALS onset than current smokers and a lower, although not statistically significant, ΔFS. All the other clinical factors (gender, BMI, FVC, El Escorial category), except the site of onset, were equally distributed across the categories. Pairwise associations between cigarettes/day, pack-years, duration of smoking, and the log-transformed ΔFS (i.e., log(ΔFS)) are reported in Figure 1. The log(ΔFS) was not correlated with the duration of smoking (*r* = 0.13, *p* = 0.406), nor it was different between the classes of cigarettes/day and pack-years. As expected, the number of pack-years was associated with the duration.

### 3.2. Alcohol Consumption

Current alcohol drinkers accounted for 147 (61.0%) of the participants, 5 patients (2.1%) were former drinkers, and 89 (36.9%) non-drinkers. No patient started drinking alcohol after their ALS diagnosis. No difference was found for the drinking status (Table 1). Table 3 shows the unadjusted comparisons of clinical variables among non-drinkers and drinkers according to the intensity (drinks/day) categories. Compared to non-drinkers, the age at ALS onset was lower in drinkers of ≤1 drink/day and higher in drinkers of >1 drink/day.

**Figure 2:**
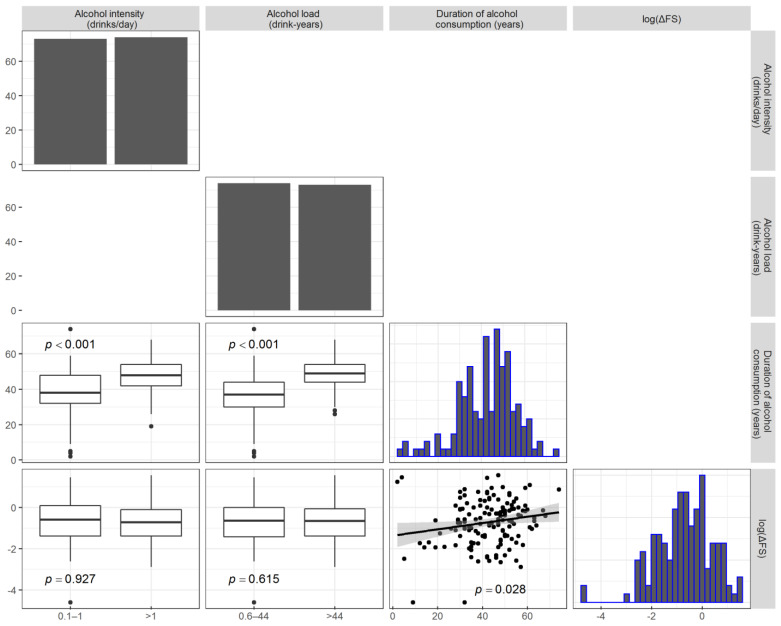
Plot matrices depicting the pairwise associations between the alcohol intensity (drinks/day), alcohol load (drink-years), duration of alcohol consumption, and log-transformed ΔFS (lower diagonal elements). Comparisons with the alcohol loads are reported as boxplots, whereas the association between the log-transformed ΔFS and duration of alcohol consumption is reported as a scatterplot with a fitted regression line. The distribution of each variable at issue is reported as a bar chart or histogram in the diagonal. Only current drinkers were considered to produce the analysis results presented here.

Former drinkers were excluded from the analysis. The disease rate of progression (median ΔFS score) was similar for all categories. All the clinical factors were equally distributed across the categories. Pairwise associations between the drinks/day, drink-years, duration of alcohol consumption, and log-transformed ΔFS were assessed, and the results are reported in Figure 2. The log(ΔFS) was weakly (but statistically significantly) correlated only with the duration of alcohol consumption (*r* = 0.18, *p* = 0.028), but not with the number of drinks/day or drink-years. As expected, the number of drink-years was associated with the duration.

Since a previous multicenter case–control study [12] found an intriguing difference in the ALS risk between patients from the Apulia region (increased) and other areas (decreased or neutral), we analyzed the subset of patients from Apulia separately (Tables S1 and S2). However, no difference in the disease progression was found for exposure to alcoholic beverages, wine alone, or smoking.

### 3.3. Predictors of ΔFS

The VIMP provided by the conditional RF algorithm that we used to detect the variables that were most associated with ΔFS suggested that diagnostic delay, age at onset, El Escorial category, and education were the covariates that explained the largest amount of the log(ΔFS) variance (Table 4). The whole RF achieved a fair goodness of fit (*R*^2^ = 0.48). Specifically, the diagnostic delay was found to be the strongest predictor of ΔFS, achieving the highest VIMP of 0.63, followed by age at onset and the El Escorial classification, whereas drinking and smoking status were at the bottom of the list (VIMP = 0).

**Figure 3:**
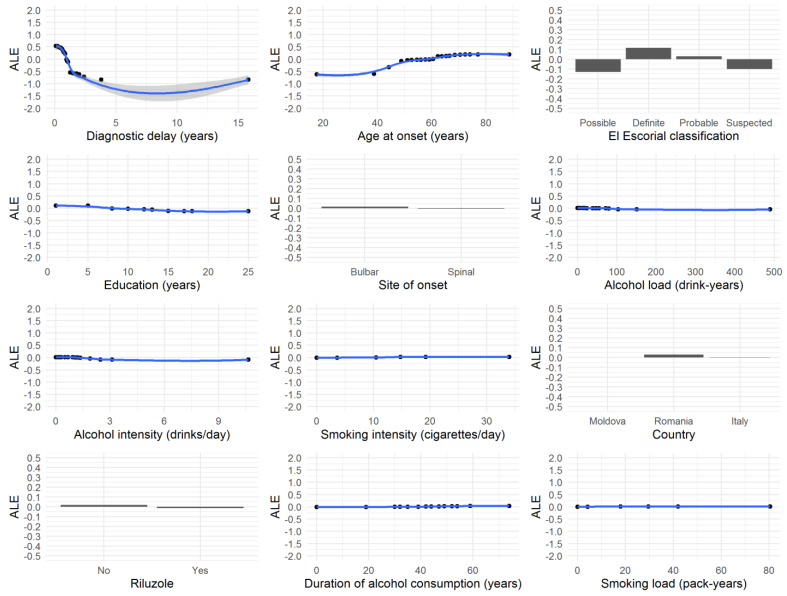
Accumulated local effects plot for each variable with variable importance > 0, as defined from the conditional random forest algorithm on log(ΔFS) values.

The accumulated local effects plot for all “important” variables (i.e., VIMP > 0) is reported in Figure 3.

The accumulated local effects can be interpreted as the change in log(ΔFS) relative to the mean for each specific value of the plotted variable of interest. As expected, the lower the diagnostic delay (close to zero), the higher the ΔFS values, and the higher the diagnostic delay (greater than 2 years), the lower the ΔFS values. In contrast, higher values of age at onset were associated with higher ΔFS values.

**Figure 4:**
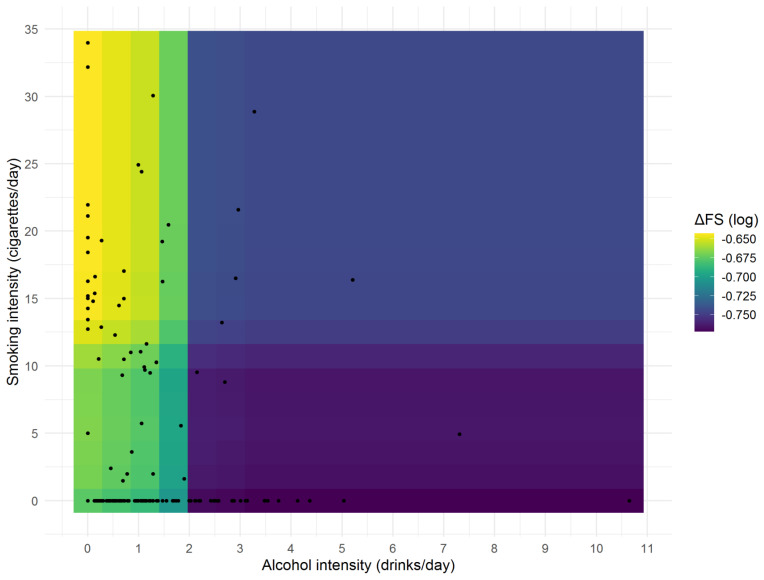
Partial dependence plot from the conditional random forest algorithm on ΔFS (log values) between smoking and alcohol intensity.

Moreover, to better understand whether smoking and alcohol intensity were jointly related with log(ΔFS), a partial dependence plot was created using a conditional RF and is shown in Figure 4.

Interestingly, from a graphical viewpoint, it seemed that the highest ΔFS was found for those who smoked more than 10 cigarettes/day but drunk less than 2 drinks/day (yellow regions), a modest ΔFS was found for those who smoked less than (or equal to) 10 cigarettes/day but drunk less than 2 drinks/day (green regions), and the lowest ΔFS was found for those who drunk at least 2 drinks/day (blue and violet regions). The association between smoking and alcohol intensity and their combination (as suggested by the partial dependence plot) with log(ΔFS) was eventually assessed in both univariable and multivariable analyses, adjusting the ANOVA models for four possible confounders (gender, age at onset, education, and diagnostic delay), both alone and in combination. Former smokers or drinkers were excluded from this analysis. The results are reported in Table 5: the ΔFS least-square means (i.e., backtransformed on the original scale) did not significantly vary across smoking and alcohol consumption groups. However, when comparing ΔFS with respect to the groups suggested by the partial dependence plot, a statistically significant difference was found in both the univariable (*p* = 0.032) and the multivariable models (all *p* < 0.05).

According to Al-Chalabi et al. [26], ALS arises as the final manifestation of a multistep process. However, the rapid progression of the pathological process after onset is an intriguing feature that remains unexplained. We were interested in the possible role of two environmental exposures in accelerating disease progression once it has started and not in their role as risk/protective factors for the onset of ALS. For this reason, we evaluated the smoking/drinking status at disease (clinical) onset by considering those who had quit smoking or drinking at least six months before onset as non-smokers/drinkers. To evaluate the possible impact of the two exposures at the earliest stage, we also included suspected ALS.

To analyze the possible role of smoking and alcohol exposures on disease progression, we divided the ΔFS into tertiles. The tertiles of the ΔFS distribution are associated with survival [17,27], thus indicating that this measure is predictive of different rates of disease progression. This was also true in our sample, where slow progressors had a younger age at disease onset, more frequent spinal onset, better FVC, and a longer diagnostic delay, which are all predictive factors for ALS progression [28,29].

Disease progression, measured using ΔFS and log(ΔFS), was only weakly correlated with the duration of alcohol consumption, but not with the alcohol drinking status, drinking intensity, or load. The age at ALS onset went in the opposite direction in the two quantity/frequency categories of drinkers compared to non-drinkers; although this observation may lend itself toward a U-shaped type of association, this must be verified in a larger sample size.

On the other hand, the age of ALS onset was lower in current smokers than non-smokers, as already observed [30], pointing to a possible effect of smoking in anticipating disease onset. Similarly, ΔFS was slightly, although not significantly, higher for smokers of >14 cigarettes/day. Indeed, as seen in Table S3, our sample only achieved 64% statistical power to detect any significant difference of log(ΔFS) means between the smoking groups (exposure).

In order to analyze a possible interaction of smoking and alcohol consumption with other clinical variables, we first ranked variables using the variable importance measure from a conditional RF and eventually performed a multivariable model. This tree-based RF algorithm is very powerful: it provides robust and “internally validated” findings without the need for the training and validation of separate datasets (i.e., RF immediately validated its decision trees in the “out of bag” observations) and, most importantly, with respect to other tree-based machine learning algorithms [31], it requires the setting of a very limited number of parameters. Clinical/demographic variables, such as diagnostic delay, age at onset, El Escorial category, and education explained the largest amount of the log(ΔFS) variance, whereas smoking and alcohol drinking retained only minor importance. After adjusting for these four variables, the multivariable analysis showed that log(ΔFS) was higher, although not significantly, for smokers of >14 cigarettes/day compared to non-smokers, and was not different among the alcohol drinking categories. However, those drinking ≥2 drinks/day, independently from their smoking status, had lower ΔFS than the two categories of drinkers and smokers, thus showing a possible interaction between the two exposures.

Taken together, the findings from this explorative study suggest possible minor roles for smoking in worsening disease progression, and conversely, for alcohol drinking. Cohort studies have been performed only for smoking, with equivocal results: smoking was identified as an independent predictor of survival in both sexes in a population registry from northwestern Italy [30], and in a U.S. study, but only in women [32]. In two other studies, smoking did not predict mortality [33,34]. Interestingly, a possible interaction between smoking and alcohol drinking as predictors of progression was found in two studies of multiple sclerosis [35,36].

This study has limitations that are intrinsic to its cross-sectional design, which prevents establishing a causal relation; however, this study design is practical for testing hypotheses in rare diseases and allows for proving associations with outcomes, if sufficiently strong, such as for smoking and severity in multiple sclerosis [35]. Furthermore, we could not evaluate the possible confounding due to unmeasured variables, such as physical activity, trauma, or diet.

On the other hand, our study does present some strengths. Selection bias was minimized because patients were consecutively enrolled and had a large spectrum of disease severity. A recall bias is unavoidable with this type of study, but patients were unaware of the study hypothesis and interviewers were blinded to clinical history and neurological status. By collecting the personal history of consumption for every single patient, we were able to study the lifelong cumulative effect of both exposures and not only the amount of exposure at the time of the interview or immediately before.

In summary, this cross-sectional multicenter study suggested only a minor role for smoking in the progression of ALS, in contrast to other neurodegenerative diseases [35,37]; the role of alcohol drinking as a possible modifier should be studied in larger samples.

## Figures and Tables

**Table 1:** Clinical and exposure variables overall and according to the tertiles of the ΔFS distribution.

Variable	Category	All (*N* = 241) *	I: Slow Progression Rate of Disease (*n* = 81)	II: Medium Progression Rate of Disease (*n* = 80)	III: Fast Progression Rate of Disease (*n* = 80)	*p*-Value	SMD
Country, *n* (%)	Italy	206 (85.5)	71 (87.7)	67 (83.8)	68 (85.0)	0.762	0.074
	Moldova/Romania	35 (14.5)	10 (12.3)	13 (16.2)	12 (15.0)		
Gender, *n* (%)	Males	145 (60.2)	53 (65.4)	44 (55.0)	48 (60.0)	0.401	0.143
	Females	96 (39.8)	28 (34.6)	36 (45.0)	32 (40.0)		
Age at recruitment (years)	Mean ± SD	62.4 ± 11.0	59.8 ± 12.3	63.6 ± 10.4	63.9 ± 9.8	0.032	0.241
Age at disease onset (years)	Mean ± SD	59.9 ± 11.8	54.6 ± 12.9	62.0 ± 10.5	63.2 ± 9.8	<0.001	0.502
Diagnostic delay (years)	Median (range)	0.9 (0.1–15.8)	1.7 (1.0–2.8)	0.8 (0.5–1.1)	0.5 (0.3–0.8)	<0.001	0.820
Education (years)	Mean ± SD	10.4 ± 4.4	11.1 ± 4.4	10.6 ± 4.3	9.5 ± 4.2	0.058	0.248
Site of onset, *n* (%)	Spinal	187 (77.6)	71 (87.7)	53 (66.2)	63 (78.8)	0.005	0.349
	Bulbar	54 (22.4)	10 (12.3)	27 (33.8)	17 (21.2)		
El Escorial categories, *n* (%)	Definite	74 (30.7)	16 (19.8)	25 (31.2)	33 (41.2)	0.014	0.460
	Possible	55 (22.8)	23 (28.4)	23 (28.7)	9 (11.2)		
	Probable	77 (32.0)	26 (32.1)	23 (28.7)	28 (35.0)		
	Suspected	35 (14.5)	16 (19.8)	9 (11.2)	10 (12.5)		
FVC, *n* (%)	<80%	88 (43.8)	20 (29.0)	32 (47.1)	36 (56.2)	0.005	0.379
	≥80%	113 (56.2)	49 (71.0)	36 (52.9)	28 (43.8)		
BMI, *n* (%)	<18.5	15 (6.2)	5 (6.2)	4 (5.0)	6 (7.5)	0.967	0.083
	18.5–24.9	121 (50.2)	42 (51.9)	40 (50.0)	39 (48.8)		
	≥25	105 (43.6)	34 (42.0)	36 (45.0)	35 (43.8)		
Riluzole, *n* (%)	Yes	129 (53.5)	41 (50.6)	47 (58.8)	41 (51.2)	0.517	0.109
	No	112 (46.5)	40 (49.4)	33 (41.2)	39 (48.8)		
Alcohol-drinking status, *n* (%)	Current drinker	147 (61.0)	49 (60.5)	52 (65.0)	46 (57.5)	0.599 ^#^	0.173
	Former drinker	5 (2.1)	1 (1.2)	3 (3.8)	1 (1.2)		
	Non-drinker	89 (36.9)	31 (38.3)	25 (31.2)	33 (41.2)		
Smoking status, *n* (%)	Current smoker	44 (18.3)	12 (14.8)	12 (15.0)	20 (25.0)	0.326 ^#^	0.226
	Former smoker	10 (4.1)	3 (3.7)	5 (6.2)	2 (2.5)		
	Non-smoker	187 (77.6)	66 (81.5)	63 (78.8)	58 (72.5)		
Age at start of smoking (years)	Mean ± SD	17.0 ± 4.2	17.4 ± 4.0	18.1 ± 5.1	15.9 ± 3.5	0.252	0.353
Age at start of drinking (years)	Mean ± SD	19.7 ± 7.4	20.0 ± 6.7	18.4 ± 5.6	21.0 ± 9.4	0.192	0.240

* Missing values were excluded from the analysis and percentages were computed out of the total number of observations. SD: standard deviation; *p*-values from ANOVA models or chi-square (with continuity correction) statistics for continuous and categorical variables, respectively. ^#^ *p*-values from Fisher exact test. SMD: standardized mean difference (i.e., the average of all possible standardized mean differences). Tertiles of ΔFS distribution were ≤0.333 (I), 0.334–0.875 (II), and >0.875 (III).

**Table 2:** Clinical variables according to the intensity of smoking during the participants’ lifetimes. Former smokers were excluded from the analysis.

Variable	Category	I: Non-Smokers (*n* = 187)	II: ≤14° Cigarettes per Day * (*n* = 21)	III: >14° Cigarettes per Day * (*n* = 23)	II vs. I (*p*-Value)	III vs. I (*p*-Value)	III vs. II (*p*-Value)
Country, *n* (%)	Italy	157 (84.0)	21 (100.0)	19 (82.6)	0.049	0.772	0.109
	Moldova/Romania	30 (16.0)	0 (0.0)	4 (17.4)			
Gender, *n* (%)	Male	103 (55.1)	16 (76.2)	18 (78.3)	0.102	0.043	1.000
	Female	84 (44.9)	5 (23.8)	5 (21.7)			
BMI (kg/m^2^), *n* (%)	<18.5	11 (5.9)	2 (9.5)	2 (8.7)	0.426	0.596	0.506
	18.5–24.9	94 (50.3)	8 (38.1)	13 (56.5)			
	≥25	82 (43.9)	11 (52.4)	8 (34.8)			
Age at recruitment (years)	Mean ± SD	63.9 ± 10.8	55.5 ± 12.1	58.3 ± 8.6	0.001	0.017	0.396
Age at disease onset (years)	Mean ± SD	61.3 ± 11.8	54.0 ± 12.4	56.6 ± 8.1	0.006	0.067	0.457
Diagnostic delay (years)^#^	Median (range)	0.9 (0.1–9.3)	0.7 (0.1–4.0)	0.6 (0.1–4.1)	0.322	0.174	0.810
Education (years)	Mean ± SD	10.5 ± 4.5	10.8 ± 4.3	10.0 ± 3.3	0.778	0.593	0.544
Site of onset, *n* (%)	Spinal	142 (75.9)	21 (100.0)	16 (69.6)	0.009	0.608	0.009
	Bulbar	45 (24.1)	0 (0.0)	7 (30.4)			
El Escorial categories, *n* (%)	Definite	61 (32.6)	6 (28.6)	4 (17.4)	0.862	0.244	0.590
	Possible	45 (24.1)	5 (23.8)	4 (17.4)			
	Probable	57 (30.5)	6 (28.6)	11 (47.8)			
	Suspected	24 (12.8)	4 (19.0)	4 (17.4)			
FVC, *n* (%)	<80%	69 (45.1)	9 (45.0)	7 (38.9)	1.000	0.803	0.752
	≥80%	84 (54.9)	11 (55.0)	11 (61.1)			
ΔFS ^#^	Median (range)	0.6 (0.0–5.3)	0.5 (0.0–2.4)	0.9 (0.1–2.7)	0.990	0.129	0.262

Missing values were excluded from the analysis and percentages were computed out of the total number of observations. SD: standard deviation; *p*-values were reported from pairwise contrasts defined in ANOVA models or Fisher’s exact test from continuous and categorical variables, respectively; ^#^ the log-transformed variable was used in the ANOVA model (because of the skewed distribution); ° median cut-off; * the smoking intensity was computed as the weighted mean of the number of cigarettes smoked per day at different age periods, with the weights equal to the smoking duration within each age period.

**Table 3:** Clinical variables according to the intensity of alcohol intake during the participants’ lifetimes. Former drinkers were excluded from the analysis.

Variable	Category	I: Non-Drinkers (*n* = 89)	II: ≤1° Drinks per Day * (*n* = 73)	III: >1° Drinks per Day * (*n* = 74)	II vs. I (*p*-Value)	III vs. I (*p*-Value)	III vs. II (*p*-Value)
Country, *n* (%)	Italy	75 (84.3)	57 (78.1)	70 (94.6)	0.319	0.045	0.004
	Moldova/Romania	14 (15.7)	16 (21.9)	4 (5.4)			
Gender, *n* (%)	Male	41 (46.1)	41 (56.2)	60 (81.1)	0.211	<0.001	0.001
	Female	48 (53.9)	32 (43.8)	14 (18.9)			
BMI (kg/m^2^), *n* (%)	<18.5	6 (6.7)	7 (9.6)	1 (1.4)	0.719	0.237	0.062
	18.5–24.9	45 (50.6)	38 (52.1)	37 (50.0)			
	≥25	38 (42.7)	28 (38.4)	36 (48.6)			
Age at recruitment (years)	Mean ± SD	62.7 ± 11.1	59.2 ± 11.5	65.3 ± 9.7	0.044	0.120	0.001
Age at disease onset (years)	Mean ± SD	60.1 ± 12.2	56.8 ± 12.3	62.9 ± 10.1	0.071	0.121	0.001
Diagnostic delay (years) ^#^	Median (range)	0.7 (0.1–9.3)	0.9 (0.1–7.5)	1.0 (0.1–15.8)	0.560	0.239	0.571
Education (years)	Mean ± SD	10.4 ± 4.5	11.0 ± 4.3	9.9 ± 4.4	0.342	0.454	0.104
Site of onset, *n* (%)	Spinal	63 (70.8)	55 (75.3)	65 (87.8)	0.596	0.012	0.058
	Bulbar	26 (29.2)	18 (24.7)	9 (12.2)			
El Escorial categories, *n* (%)	Definite	31 (34.8)	14 (19.2)	27 (36.5)	0.008	0.576	0.005
	Possible	16 (18.0)	19 (26.0)	19 (25.7)			
	Probable	25 (28.1)	34 (46.6)	16 (21.6)			
	Suspected	17 (19.1)	6 (8.2)	12 (16.2)			
FVC, *n* (%)	<80%	30 (41.1)	24 (43.6)	32 (46.4)	0.857	0.612	0.856
	≥80%	43 (58.9)	31 (56.4)	37 (53.6)			
ΔFS ^#^	Median (range)	0.6 (0.0–5.3)	0.6 (0.0–4.3)	0.5 (0.1–4.8)	0.795	0.720	0.926

Missing values were excluded from the analysis and percentages were computed out of the total number of observations. SD: standard deviation; *p*-values were reported from the pairwise contrasts defined in ANOVA models or Fisher exact test from continuous and categorical variables, respectively; ^#^ the log-transformed variable was used in the ANOVA model (because of the skewed distribution); ° median cut-off; * the drinking intensity was computed as the weighted mean number of standard alcoholic units per day at different age periods, with the weights equal to the number of years spent drinking (i.e., drinking duration) within each age period for all types of beverages.

**Table 4:** Variable importance (VIMP) and relative variable importance (RVIMP) values from conditional random forest algorithm (100,000 trees) of each candidate’s clinical, demographical, pathological, treatment, and smoking/alcohol consumption variables for explaining the variability of the log(ΔFS) values. Variables are ranked from the most to the least important (rank).

Variable	Conditional VIMP	Conditional RVIMP
Diagnostic delay	0.6302	100.0%
Age at onset	0.1680	26.7%
El Escorial classification	0.0413	6.6%
Education	0.0278	4.4%
Site of onset	0.0072	1.1%
Alcohol load (drink-years)	0.0043	0.7%
Alcohol intensity (drinks/day)	0.0043	0.7%
Smoking intensity (cigarettes/day)	0.0016	0.3%
Country	0.0014	0.2%
Riluzole	0.0007	0.1%
Alcohol duration	0.0005	0.1%
Smoking load (pack-years)	0.0002	0.0%
BMI	0.0000	0.0%
Smoking duration	0.0000	0.0%
Alcohol drinking status	0.0000	0.0%
Smoking status	0.0000	0.0%
Gender	0.0000	0.0%

The VIMP of a specific variable is the sum of the decrease in prediction error values (of log(ΔFS)) when a tree of the forest splits due to that variable, whereas RVIMP is the VIMP divided by the highest VIMP value such that values are bounded between 0 and 1 (or between 0 and 100%).

**Table 5:** Effect of smoke and alcohol consumption during the participants’ lifetimes on ΔFS: results from the ANOVA models. Former consumers were excluded from the analysis.

		Estimated ΔFS Means (95% CI) ^#^			
Exposure (Groups)	Confounders	Group 1	Group 2	Group 3	*p*-Value *
Smoke intensity 1: 0 cigarettes/day 2: 1.5–14 cigarettes/day 3: >14 cigarettes/day	None	0.49 (0.42–0.57)	0.49 (0.31–0.78)	0.71 (0.45–1.10)	0.313
	Age at onset	0.47 (0.41–0.54)	0.61 (0.40–0.95)	0.80 (0.53–1.22)	0.255
	Gender	0.49 (0.42–0.58)	0.51 (0.32–0.81)	0.73 (0.47–1.16)	0.313
	Education	0.49 (0.42–0.57)	0.49 (0.31–0.78)	0.69 (0.44–1.07)	0.303
	Diagnostic delay (log)	0.51 (0.44–0.57)	0.44 (0.30–0.64)	0.60 (0.42–0.87)	0.174
	Age at onset + gender	0.47 (0.41–0.55)	0.65 (0.42–1.01)	0.86 (0.56–1.31)	0.252
	Age at onset + education	0.47 (0.41–0.54)	0.61 (0.39–0.94)	0.79 (0.52–1.20)	0.255
Alcohol intensity 1: 0 drinks/day 2: 0.1–1 drinks/day 3: >1 drinks/day	None	0.52 (0.41–0.65)	0.50 (0.39–0.64)	0.49 (0.38–0.63)	0.932
	Age at onset	0.52 (0.42–0.64)	0.56 (0.44–0.71)	0.44 (0.35–0.55)	0.921
	Gender	0.52 (0.41–0.65)	0.50 (0.39–0.64)	0.51 (0.39–0.66)	0.932
	Education	0.52 (0.41–0.65)	0.51 (0.40–0.66)	0.48 (0.37–0.61)	0.930
	Diagnostic delay (log)	0.49 (0.41–0.59)	0.50 (0.41–0.61)	0.52 (0.42–0.64)	0.899
	Age at onset + gender	0.51 (0.42–0.64)	0.56 (0.44–0.71)	0.46 (0.36–0.58)	0.921
	Age at onset + education	0.52 (0.42–0.64)	0.56 (0.44–0.71)	0.44 (0.35–0.55)	0.921
RF classification 1: 0–1 drinks/day and >10 cigarettes/day 2: 0–1 drinks/day and ≤10 cigarettes/day 3: ≥2 drinks/day	None	0.73 (0.49–1.10)	0.51 (0.43–0.60)	0.35 (0.24–0.51)	0.032
	Age at onset	0.88 (0.61–1.27)	0.50 (0.43–0.59)	0.33 (0.23–0.46)	0.016
	Gender	0.75 (0.50–1.14)	0.51 (0.43–0.60)	0.36 (0.24–0.54)	0.032
	Education	0.73 (0.49–1.08)	0.52 (0.44–0.61)	0.34 (0.23–0.49)	0.028
	Diagnostic delay (log)	0.61 (0.44–0.85)	0.51 (0.45–0.59)	0.40 (0.30–0.55)	0.006
	Age at onset + gender	0.92 (0.63–1.34)	0.50 (0.43–0.59)	0.35 (0.24–0.50)	0.016
	Age at onset + education	0.87 (0.60–1.26)	0.51 (0.44–0.59)	0.32 (0.23–0.46)	0.016

* *p*-value from ANOVA model (type 3 test); ^#^ log-transformed ΔFS values were used in the ANOVA models and their means were backtransformed to their original scales; random forest classification: groups defined by looking at the partial dependence plot that was created from the conditional random forest.

## Data Availability

The data presented in this study are available on request from the corresponding author. The data are not publicly available due to ethical and privacy restrictions.

## Supplementary Materials

The following are available online at https://www.mdpi.com/article/10.3390/life11040352/s1.

## References

[1] Creemers H., Grupstra H., Nollet F., van den Berg L.H., Beelen A. (2015). Prognostic factors for the course of functional status of patients with ALS: A systematic review. J. Neurol..

[2] Krewski D., Barakat-Haddad C., Donnan J., Martino R., Pringsheim T., Tremlett H., van Lieshout P., Walsh S.J., Birkett N.J., Gomes J. (2017). Determinants of neurological disease: Synthesis of systematic reviews. Neurotoxicology.

[3] Belbasis L., Bellou V., Evangelou E. (2016). Environmental Risk Factors and Amyotrophic Lateral Sclerosis: An Umbrella Review and Critical Assessment of Current Evidence from Systematic Reviews and Meta-Analyses of Observational Studies. Neuroepidemiology.

[4] Kamel F., Umbach D.M., Munsat T.L., Shefner J.M., Sandler D.P. (1999). Association of cigarette smoking with amyotrophic lateral sclerosis. Neuroepidemiology.

[5] Gallo V., Bueno-De-Mesquita H.B., Vermeulen R., Andersen P.M., Kyrozis A., Linseisen J., Kaaks R., Allen N.E., Roddam A.W., Boshuizen H.C. (2009). Smoking and risk for amyotrophic lateral sclerosis: Analysis of the EPIC cohort. Ann. Neurol..

[6] Wang M.D., Little J., Gomes J., Cashman N.L., Krewski D. (2017). Identification of risk factors associated with onset and progression of amyotrophic lateral sclerosis using systematic review and meta-analysis. NeuroToxicology.

[7] Peters S., Visser A.E., D’Ovidio F., Vlaanderen J., Portengen L., Beghi E., Chio A., Logroscino G., Hardiman O., Pupillo E. (2020). Effect modification of the association between total cigarette smoking and ALS risk by intensity, duration and time-since-quitting: Euro-MOTOR. J. Neurol. Neurosurg. Psychiatry.

[8] Opie-Martin S., Jones A., Iacoangeli A., Al-Khleifat A., Oumar M., Shaw P.J., Shaw C.E., Morrison K.E., Wootton R.E., Davey-Smith G. (2020). UK case control study of smoking and risk of amyotrophic lateral sclerosis. Amyotroph. Lateral Scler. Front. Degener..

[9] Yu X., Wang T., Chen Y., Shen Z., Gao Y., Xiao L., Zheng J., Zeng P. (2020). Alcohol Drinking and Amyotrophic Lateral Sclerosis: An Instrumental Variable Causal Inference. Ann. Neurol..

[10] E M., Yu S., Dou J., Jin W., Cai X., Mao Y., Zhu D., Yang R. (2016). Association between alcohol consumption and amyotrophic lateral sclerosis: A meta-analysis of five observational studies. Neurol. Sci..

[11] Peng B., Yang Q., BJoshi R., Liu Y., Akbar M., Song B.J., Zhou S., Wang X. (2020). Role of Alcohol Drinking in Alzheimer’s Disease, Parkinson’s Disease, and Amyotrophic Lateral Sclerosis. Int. J. Mol. Sci..

[12] D’Ovidio F., Rooney J.P.K., Visser A.E., Manera U., Beghi E., Logroscino G., Vermeulen R.C.H., Veldink J.H., van den Berg L.H., Hardiman O. (2019). Association between alcohol exposure and the risk of amyotrophic lateral sclerosis in the Euro-MOTOR study. J. Neurol. Neurosurg. Psychiatry.

[13] Waubant E., Lucas R., Mowry E., Graves J., Olsson T., Alfredsson L., Langer-Gould A. (2019). Environmental and genetic risk factors for MS: An integrated review. Ann. Clin. Transl. Neurol..

[14] von Elm E., Altman D.G., Egger M., Pocock S.J., Gøtzsche P.C., Vandenbroucke J.P. (2007). STROBE Initiative. The Strengthening the Reporting of Observational Studies in Epidemiology (STROBE) statement: Guidelines for reporting observational studies. Lancet.

[15] Brooks B.R. (1994). El Escorial World Federation of Neurology criteria for the diagnosis of amyotrophic lateral sclerosis. Subcommittee on Motor Neuron Diseases/Amyotrophic Lateral Sclerosis of the World Federation of Neurology Research Group on Neuromuscular Diseases and the El Escorial’ Clinical limits of amyotrophic lateral sclerosis’ workshop contributors. J. Neurol. Sci..

[16] Cedarbaum J.M., Stambler N., Malta E., Fuller C., Hilt D., Thurmond B., Nakanishi A. (1999). The ALSFRS-R: A revised ALS functional rating scale that incorporates assessments of respiratory function. J. Neurol. Sci..

[17] Kimura F., Fujimura C., Ishida S., Nakajima H., Furutama D., Uehara H., Shinoda K., Sugino M., Hanafusa T. (2006). Progression rate of ALSFRS-R at time of diagnosis predicts survival time in ALS. Neurology.

[18] Riboli E., Hunt K.J., Slimani N., Ferrari P., Norat T., Fahey M., Charrondière U.R., Hémon B., Casagrande C., Vignat J. (2002). European Prospective Investigation into Cancer and Nutrition (EPIC): Study populations and data collection. Public HealthNutr..

[19] Ferrari P., Jenab M., Norat T., Moskal A., Slimani N., Olsen A., Tjønneland A., Overvad K., Jensen M.K., Boutron-Ruault M.-C. (2007). Lifetime and baseline alcohol intake and risk of colon and rectal cancers in the European prospective investigation into cancer and nutrition (EPIC). Int. J. Cancer..

[20] Centers for Disease Control (2002). Cigarette smoking among adults–United States, 2000. Morb. Mortal. Wkly. Rep..

[21] Istituto Nazionale di Ricerca per gli Alimenti e la Nutrizione (INRAN) (2003). Linee Guida per Una Sana Alimentazione Italiana. https://www.crea.gov.it/documents/59764/0/lineeguida_intro.pdf/9b1a2730-2306-5c15-92f6-bab936eaf113?t=1551861382106.

[22] Landis J.R., Koch G.C. (1977). The measurement of observer agreement for categorical data. Biometrics.

[23] Bartko J.J. (1966). The intraclass correlation coefficient as a measure of reliability. Psychol. Rep..

[24] Strobl C., Boulesteix A.-L., Kneib T., Augustin T., Zeileis A. (2008). Conditional variable importance for random forests. BMC Bioinform..

[25] Apley D.W., Zhu J. (2016). Visualizing the effects of predictor variables in black box supervised learning models. arXiv.

[26] Al-Chalabi A., Calvo A., Chio A., Colville S., Ellis C.M., Hardiman O., Heverin M., Howard R.S., Huisman M.H.B., Keren N. (2014). Analysis of amyotrophic lateral sclerosis as a multistep process: A population-based modelling study. Lancet Neurol..

[27] Labra J., Menon P., Byth K., Morrison S., Vucic S. (2016). Rate of disease progression: A prognostic biomarker in ALS. J. Neurol. Neurosurg. Psychiatry.

[28] Chiò A., Logroscino G., Hardiman O., Swingler R., Mitchell D., Beghi E., Traynor B.G., Eurals Consortium (2009). Prognostic factors in ALS: A critical review. Amyotroph. Lateral Scler..

[29] Westeneng H.J., Debray T.P.A., Visser A.E., van Eijk R.P.A., Rooney J.P.K., Calvo A., Martin S., McDermott C.J., Thompson A.G., Pinto S. (2018). Prognosis for patients with amyotrophic lateral sclerosis: Development and validation of a personalised prediction model. Lancet Neurol..

[30] Calvo A., Canosa A., Bertuzzo D., Cugnasco P., Solero L., Clerico M., De Mercanti S., Bersano E., Cammarosano S., Ilardi A. (2016). Influence of cigarette smoking on ALS outcome: A population-based study. J. Neurol. Neurosurg. Psychiatry.

[31] Hothorn T., Jung H.H. (2014). RandomForest4Life: A Random Forest for predicting ALS disease progression. Amyotroph. Lateral Scler. Front. Degener.

[32] Alonso A., Logroscino G., Jick S.S., Hernán M.A. (2010). Association of smoking with amyotrophic lateral sclerosis risk and survival in men and women: A prospective study. BMC Neurol..

[33] del Aguila M.A., Longstreth W.T., McGuire V., Koepsell T.D., van Belle G. (2003). Prognosis in amyotrophic lateral sclerosis: A population-based study. Neurology.

[34] Paillisse C., Lacomblez L., Dib M., Bensimon G., Garcia-Acosta S., Meininger V. (2005). Prognostic factors for survival in amyotrophic lateral sclerosis patients treated with riluzole. Amyotroph. Lateral Scler. Other Motor. Neuron. Disord..

[35] Ivashynka A., Copetti M., Naldi P., D’Alfonso S., Leone M.A. (2019). The Impact of Lifetime Alcohol and Cigarette Smoking Loads on Multiple Sclerosis Severity. Front. Neurol..

[36] Hedstrom A.K., Hillert J., Olsson T., Alfredsson L. (2014). Alcohol as a modifiable lifestyle factor affecting multiple sclerosis risk. JAMA Neurol..

[37] Belvisi D., Pellicciari R., Fabbrini G., Tinazzi M., Berardelli A., Defazio G. (2020). Modifiable risk and protective factors in disease development, progression and clinical subtypes of Parkinson’s disease: What do prospective studies suggest?. Neurobiol. Dis..

