# Supplementary material for: The Impact of Lifetime Alcohol and Cigarette Smoking Loads on Amyotrophic Lateral Sclerosis Progression: A Cross-Sectional Study
Source: Life (Basel). 2021 Apr 17;11(4):352. doi: 10.3390/life11040352 (PMC8072690; doi:10.3390/life11040352)
Supplement: Supplementary file 1 [file life-11-00352-s001.zip › life-1128781-supplementary.pdf]

Table S1. Alcohol and smoking status overall and according to the tertiles of ΔFS distribution among patients in Apulia region.

**Variable**	**Category**	**All (N = 57)**	**I: Slow progression rate of disease (N = 21)**	**II: Medium progression rate of disease (N = 13)**	**III: Fast progression rate of disease (N = 23)**	***p*-value**	**SMD**
Alcoholic drinking status - N(%)	Current drinker	35 (61.4)	12 (57.1)	9 (69.2)	14 (60.9)	0.453[#]	0.385
	Former drinker	1 (1.8)	0 (0.0)	1 (7.7)	0 (0.0)		
	Non-drinker	21 (36.8)	9 (42.9)	3 (23.1)	9 (39.1)		
Wine drinking status - N(%)	Current drinker	33 (57.9)	12 (57.1)	8 (61.5)	13 (56.5)	0.194[#]	0.469
	Former drinker	2 (3.5)	0 (0.0)	2 (15.4)	0 (0.0)		
	Non-drinker	22 (38.6)	9 (42.9)	3 (23.1)	10 (43.5)		
Smoking habits - N(%)	Current smoker	10 (17.5)	4 (19.0)	4 (30.8)	2 (8.7)	0.241[#]	0.524
	Former smoker	2 (3.5)	0 (0.0)	1 (7.7)	1 (4.3)		
	Non-smoker	45 (78.9)	17 (81.0)	8 (61.5)	20 (87.0)		

Patients represent a subgroup of all 241 ALS patients, with residency in Apulia. Tertiles of ΔFS distribution were ≤ 0.333 (I); 0.334 – 0.875 (II); >0.875 (III)

Table S2. ΔFS distribution according to alcohol load (during lifetime) in Apulia ALS patients. Former drinkers were excluded from the analysis.

Variable	Statistic	All (N=56)	I: Non-drinkers (N=21)	II: ≤1° drinks per day* (N=14)	III: >1° drinks per day* (N=21)	II vs. I (p-value)	III vs. I (p-value)	III vs. II (p-value)
ΔFS#	Median (range)	0.68 [0.00-5.33]	0.64 [0.02-5.33]	0.65 [0.00-4.33]	0.72 [0.08-4.20]	0.921	0.781	0.881

Patients represent a subgroup of all 241 ALS patients, with residency in Apulia.

SD: standard deviation; p-values were reported from pairwise contrasts defined in ANOVA models; #log-transformed variable was used in the ANOVA model (because of skewed distribution); °Median cut-off; *The drinking intensity was computed as the weighted mean number of standard alcoholic units per day at different age periods with weights equal to the number of years spent drinking (i.e. drinking duration) within each age period for all type of beverages

Table S3. Details for power calculation to detect a statistically significant ($p<0.05$) difference of log-ΔFS means among smoke groups (i.e. non-smokers vs. light vs.heavy smokers) using a one-way ANOVA model. Former smokers are not considered in the present analysis.

		log-ΔFS	
**Smoke groups**	**N**	**Mean**	**SD**
Non-smokers	187	-0.714	1.067
≤14 cigarettes per day	21	-0.717	1.344
>14 cigarettes per day	23	-0.349	0.991
Overall	231	-0.678	1.088

$$\mathbf{SDm} = \sqrt{\frac{[(-0.714)-(-0.678)]^2}{3} + \frac{[(-0.717)-(-0.678)]^2}{3} + \frac{[(-0.349)-(-0.678)]^2}{3}} \approx 0.19$$

Given the groups sample size of 187, 21 and 23 subjects and under the assumption that the log-ΔFS's SD of 1.1 was the same within each group, this sample achieved 80% of statistical power (i.e. 1- type II error) to detect a SDm of 0.23 as statistically significant, using a one-way ANOVA model, having fixed a type I error of 5%. Because the observed SDm was lower than the expected, we found that the actual statistical power was 64%.

N: number of subjects; SD: standard deviation of log-ΔFS; SDm: standard deviation of log-ΔFS means
